# Dairy cows value access to pasture as highly as fresh feed

**DOI:** 10.1038/srep44953

**Published:** 2017-03-23

**Authors:** Marina A. G. von Keyserlingk, Andressa Amorim Cestari, Becca Franks, Jose A. Fregonesi, Daniel M. Weary

**Affiliations:** 1 Animal Welfare Program, Faculty of Land and Food Systems, University of British Columbia, 2357 Main Mall, Vancouver, BC, Canada; 2 Universidade Estadual de Londrina, Parana, CEP-86051-990, Brazil

## Abstract

Many dairy cows in the developed world are now housed exclusively indoors with fewer than 5% of the 10 million lactating cows in the United States having access to pasture during the grazing season. Indoor housing systems are designed to meet biological needs for food, water, hygiene, and shelter, but surveys of public and farmer opinion suggest that people think that pasture access is also important for the well-being of dairy cows. To determine if pasture access is important to the cows themselves, we investigated to what extent cows will work to access pasture (by pushing on a weighted gate), and compared it to the motivation to access fresh feed. Cows worked at least as hard to access pasture as they did to access the fresh feed and worked hardest for outdoor access in the evening hours. Echoing public views on what allows for a good life for cattle, these results show that cows are highly motivated for outdoor access.

Previous work has shown that dairy cows typically choose to remain indoors during the day, particularly when temperature and humidity are high, but will spend most of their time on pasture at night if provided the opportunity^1^. These results show a preference for outdoor access at night, but do not tell us the strength of this preference. Some preferences may be trivial, making it difficult to draw strong inferences about the welfare consequences of pasture access based on these results. Operant responses can be used to experimentally assess motivation for access to a specific resource such as pasture. Once animals have learned to perform an operant task to obtain access to a resource, the ‘work’ required for each access can be increased. Resources that are very important to the animal show a relatively inelastic demand, a concept used frequently by economists and adapted by Dawkins (1983) for the study of animal welfare^2^. Rewards are considered to have inelastic demand if animals are willing to work to maintain a given level of the reward even when the costs (e.g. weight pushed, distance travelled, pain endured) increase. Resources of less value, i.e. luxuries, have elastic demand: as the costs increase, animals stop working to access the reward. Thus, the relative importance of a reward can be assessed with the elasticity of the demand curve. The reservation price or maximum price paid, i.e., the highest effort that the animals complete, is an additional way of estimating the value that the animal attaches to the resource^3^.

By relying on decision making (the choice of working or not working for the resource), these measures of motivation can distinguish between weak and fleeting preferences that may have little consequence for animal welfare, and resources and experiences that the animal considers to be more valuable. For example, to investigate feeding motivation, some authors^4^ measured the maximum distance walked to obtain food when cows were exposed to different levels of feed deprivation, and found that cows were willing to walk longer distances to access feed after longer periods of feed deprivation. Weighted doors (that animals push open to access a reward have also been used to study motivation for access to specific resources^5^, including access to a dust bathing area^6^ and a nest box^7^ by chickens, and access to a water bath by farmed mink^8^. This experimental paradigm is based on the concept that animals will be willing to push heavier weights to access more important resources.

The objective of this experiment was to assess cows’ motivation to access pasture and compare it to the motivation to access fresh food immediately after milking, when cows are most motivated to eat^9^ and thus a gold-standard for inelastic demand^10^. We hypothesized that cows would work to access pasture, but that the motivation for pasture access would be less than that for fresh feed after milking. We also predicted that cows would push more weight in the evening than during the day, consistent with the preference data showing stronger preferences for outdoor access at night.

## Materials and Methods

We used 22 pregnant, late lactation Holstein cows averaging (mean ± SD) 221 ± 14.4 days in milk, producing on average 30.0 ± 3.8 L of milk/day, 1.4 ± 0.7 parity, weighing 648 ± 93.3 kg, and body condition score of 3.0 ± 0.4 (scored from 1 to 5)^11^. The study was approved by the University of British Columbia’s Animal Care Committee (#A10-0162) and cared for according to the guidelines outlined by the Canadian Council of Animal Care^12^.

Cows were tested in two phases. In Phase 1 we used two indoor pens fitted with lying stalls, a designated feeding area and fresh drinking water. Cows were milked twice daily at approximately 0630 and 1700 h. Cows were offered fresh feed in the form of a total mixed ration (TMR) ad libitum, delivered daily at approximately 0630 h and 1600 h. The TMR consisted of 21.8% corn silage, 18.6% grass silage, 10.3% alfalfa hay and 49.3% concentrate mash, on a dry matter basis to *ad libitum* intake. The TMR was composed of 48.2% dry matter on average, with 18.5% crude protein, 31.6% neutral detergent fibre and 18.9% acid detergent fibre.

A weighted push-gate (see supplementary Video) was used to assess motivation to access fresh feed. We trained cows twice daily to open the push-gate placed between two adjacent pens (one without feed) and the other with fresh feed. To obtain access to the feed cows were required to open the push-gate in the adjacent pen. Cows were considered trained when the gate was successfully pushed open in four consecutive training sessions. Cows were allowed to access the gate starting 1.5 h after each milking. An additional 7 kg of weight was added daily to the initial 7 kg training weight until cows did not perform the task for two consecutive sessions. Cows were given 2.5 h to complete the task. The maximum weight pushed for access to the feed (i.e. maximum price paid) was recorded for each individual cow. Behaviors were recorded using a surveillance camera (Panasonic, CCTV, WV-BP334, Osaka, Japan) installed 3 m over the push gate.

The procedure for Phase 2 was identical except the push gate was positioned between the indoor pen and the pasture. To ensure that cows were acclimated to the specific pasture used in this experiment, all cows were kept on this pasture for 5 d before testing. The distance between the pasture and the barn entrance was 12 m. All cows had access to the same fresh TMR described in Phase 1 inside the barn throughout the entire study. As in Phase 1, the push gate was fitted with an intial weight of 7 kg and this weight was increased by 7 kg every 24 hours. The test stopped when no cow within the group exited the gate for two consecutive days.

## Results

**Figure 1:**
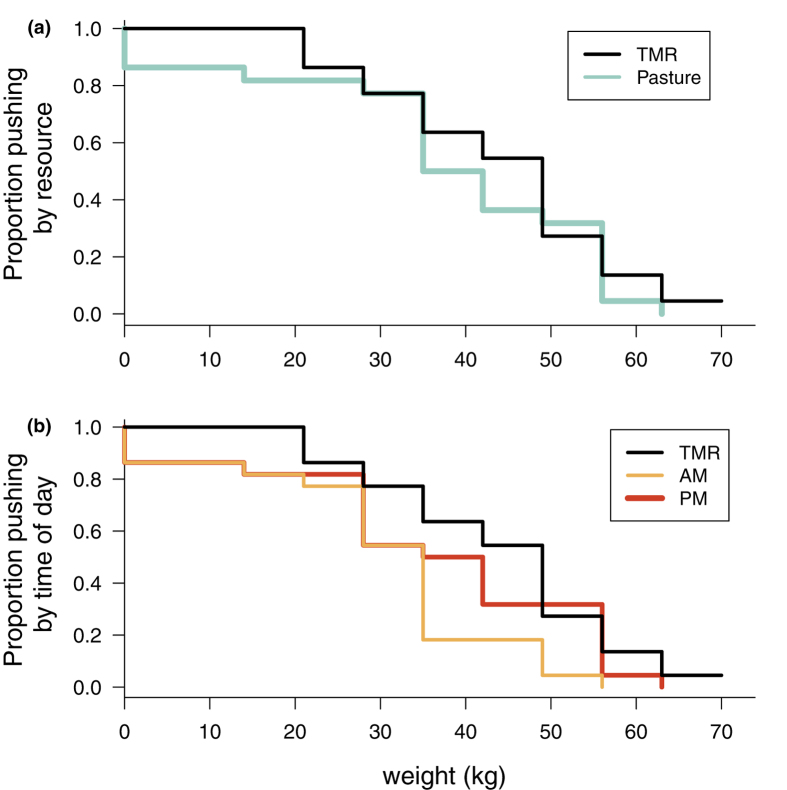
Survival plots of cows’ willingness to work for types of resources. (**a**) Overall, the motivation to access pasture (AM & PM combined) was as strong as it was to access food after 1.5 hours of fasting (log-rank test for difference between survival distributions: P > 0.4). (**b**) The motivation to access pasture was stronger in the afternoon (log-rank test PM vs TMR: P > 0.3) than in the morning (log-rank test AM vs TMR: P < 0.01).

The majority of cows (59%) pushed just as hard or harder to access pasture as they did to access the TMR (binomial test P > 0.52). Modeling the number of cows who were willing to push at each weight with a survival analysis allowed us to estimate a demand curve for the group, with steeper survival curves indicating more elastic demand (less valuable resources). Survival analysis (log-rank: P > 0.42; Fig. 1a) and reservation price (mean max weight [SEM] in kg: pasture = 31.18 [3.82] and TMR = 36.91 [3.06]; paired t(18) = 1.58, P > 0.12) both failed to detect a difference in motivation for the two resources. After the evening milking, cows pushed harder for access to pasture than in the morning (binomial test P = 0.05), with survival analysis (log-rank test PM vs TMR: P > 0.3; log-rank test AM vs TMR: P < 0.01; Fig. 1b) and reservation price (mean max weight [SEM] in kg: afternoon = 29.27 [3.90] and morning = 23.55 [3.00]; paired t(18) = 2.08, P < 0.05) both showing higher motivation for pasture in the evening.

## Discussion

Access to fresh feed was considered our gold standard given that cows are known to be highly motivated to access fresh feed after milking^9^; we expected that the weight pushed to access the feed would be close to the maximum amount that cows were able to push. We had expected pasture to be valuable to the cows, but not as valuable as access to the fresh feed, but instead found no difference in motivation to access the two resources.

Cows pushed harder to access pasture after the evening milking; this result is consistent with earlier results showing that preference for access to pasture is stronger at night^1^,^13^, likely because solar heating can make cows uncomfortably hot if they are outdoors during the heat of the day^14^. We expect that this greater motivation for evening access would be less pronounced if cows were tested in the winter when radiant heating is less likely to cause discomfort.

## Conclusion

The results of the current study show that dairy cows are as motivated to access pasture as they are to eat fresh feed two hours after milking. As cows had free access to fresh feed inside the barn when tested for motivation to access pasture, our results suggest that motivation to access pasture was not driven by hunger, but rather motivation to be outside (and to engage in behaviors associated with outdoor access, including grazing). Further research could investigate the nature of this motivation by varying the outdoor conditions (e.g. providing grazing opportunities vs. outdoor access only). Addressing public concerns over animal welfare will require better data on the animals under our care—data that include not only the motivation for material outcomes such as food and shelter, but also the motivation to engage in behavioral processes for their own sake.

## Additional Information

**How to cite this article:** von Keyserlingk, M. A. G. *et al*. Dairy cows value access to pasture as highly as fresh feed. *Sci. Rep.* **7**, 44953; doi: 10.1038/srep44953 (2017).

**Publisher's note:** Springer Nature remains neutral with regard to jurisdictional claims in published maps and institutional affiliations.

## Supplementary Material

Supplementary Information

Supplementary Video 1

Supplementary Dataset
