# Supplementary material for: Dairy cows value access to pasture as highly as fresh feed
Source: Sci Rep. 2017 Mar 23;7:44953. doi: 10.1038/srep44953 (PMC5362966; doi:10.1038/srep44953)
Supplement: Supplementary Information [file srep44953-s1.docx]

Dairy cows value access to pasture as highly as fresh feed

Marina A.G. von Keyserlingk^*^, Andressa Amorim Cestari **^*,‡^,** Becca A. Franks**^*^** , Jose Fregonesi^‡^ and Daniel M. Weary^*^

^*^Animal Welfare Program, Faculty of Land and Food Systems, University of British Columbia, 2357 Main Mall, Vancouver, BC CANADA, ‡Universidade Estadual de Londrina, Parana, CEP-86051-990, Brazil

Corresponding author email: marina.vonkeyserlingk@ubc.ca

Video 1. Video shows the weighted pushgate that was used to test the cows’ willingness to work for resources (feed or pasture). Title of manuscript: Dairy cows value access to pasture as highly as fresh feed. Authors: Marina A.G. von Keyserlingk, Andressa Amorim Cestari , Becca A. Franks, Jose Fregonesi and Daniel M. Weary.
